# Outbreeding management offers the promise of genetic rescue for an endangered lizard

**DOI:** 10.1093/cz/zoae003

**Published:** 2024-02-27

**Authors:** Guannan Wen, Hongxin Xie, Shuyi Luo, Chunsheng Yang, Xianwu Tang, Yibo Hu, Weiguo Du

**Affiliations:** Key Laboratory of Animal Ecology and Conservation Biology, Institute of Zoology, Chinese Academy of Sciences, 1# Beichen West Road, Chaoyang District, Beijing, 100101, P.R. China; Key Laboratory of Animal Ecology and Conservation Biology, Institute of Zoology, Chinese Academy of Sciences, 1# Beichen West Road, Chaoyang District, Beijing, 100101, P.R. China; University of Chinese Academy of Sciences, 19A# Yuquan Road, Shijingshan District, Beijing, 100049, P.R. China; Daguishan National Nature Reserve for Crocodile Lizards, 80# Jianshe West Road, Babu District, Hezhou, 542800, Guangxi, P.R. China; Daguishan National Nature Reserve for Crocodile Lizards, 80# Jianshe West Road, Babu District, Hezhou, 542800, Guangxi, P.R. China; Daguishan National Nature Reserve for Crocodile Lizards, 80# Jianshe West Road, Babu District, Hezhou, 542800, Guangxi, P.R. China; Key Laboratory of Animal Ecology and Conservation Biology, Institute of Zoology, Chinese Academy of Sciences, 1# Beichen West Road, Chaoyang District, Beijing, 100101, P.R. China; Key Laboratory of Animal Ecology and Conservation Biology, Institute of Zoology, Chinese Academy of Sciences, 1# Beichen West Road, Chaoyang District, Beijing, 100101, P.R. China

**Keywords:** conservation genetics, ex situ conservation, genetic rescue, heterosis, inbreeding

## Abstract

Inbreeding and loss of genetic diversity increase the extinction risk of small isolated populations. Genetic rescue by augmenting gene flow is a powerful means for the restoration of lost genetic variation. In this study, we used multigenerational pedigrees and neutral genetic markers to assess the consequences of outbreeding management in the Chinese and Vietnamese populations of the endangered crocodile lizard, *Shinisaurus crocodilurus*. Compared with the purebred Chinese population, the outbreeding population exhibited greater molecular genetic variation and a 3-fold larger population size. Moreover, the first-generation hybrids had a longer lifespan than purebreds, suggesting that outbreeding depression did not occur, but the long-term fitness effect of outbreeding needs to be further evaluated. Our study provides valuable insights into the potential for genetic rescue in the endangered crocodile lizard, emphasizing the importance of an evidence-based genetic management approach to address the risks of inbreeding and outbreeding depression in threatened populations.

The deleterious effect of inbreeding and associated loss of genetic diversity are recognized as major contributors to the increased extinction risk for species with small and fragmented populations (Frankham et al. 2002; Spielman et al. 2004; O’Grady et al. 2006). A variety of natural and experimental studies have shown that immigration of genetically divergent individuals can alleviate inbreeding depression and boost fitness for small, inbred, at-risk populations. This is known as genetic rescue (Tallmon et al. 2004; Hedrick et al. 2011; Whiteley et al. 2015). Genetic rescue stands out as one of the most powerful means for conserving small natural populations, given that immigration has the potential to augment the fitness of local populations (Tallmon et al. 2004; Frankham 2015; Whiteley et al. 2015). Perhaps the best-known case of genetic rescue is that of the Florida Panther (*Puma concolor coryi*). This previously highly inbred population exhibited significant improvements in various fitness-related traits, molecular genetic variation, and abundance after the translocation of 8 panthers from Texas (Pimm et al. 2006; Johnson et al. 2010). Recent instances, such as Coho salmon (Pregler et al. 2023) and Panamanian harlequin frogs (Byrne et al. 2021), further highlight the potential benefits of genetic rescue. Despite these successes, the augmentation of gene flow remains infrequent in conservation programs (Frankham et al. 2017; Love Stowell et al. 2017). This reluctance is primarily due to concerns surrounding outbreeding depression (Edmands 2007), the possible loss of unique local adaptations (Johnson et al. 2010), and uncertainties about the most effective approaches for establishing long-term, sustainable, and resilient populations (Love Stowell et al. 2017; Ralls et al. 2018, 2020; Bell et al. 2019). However, numerous meta-analyses have demonstrated the advantages of outcrossing and have provided insights into when and how to address its potential adverse effects (Frankham et al. 2011; Frankham 2015, 2016; Ralls et al. 2020). With the ongoing extinction crisis, calls for broader use of augmented gene flow in small, inbred populations of endangered species are becoming increasingly louder (Ralls et al. 2018; Bell et al. 2019; Fitzpatrick et al. 2020). Deliberate efforts to experimentally examine genetic rescue and outbreeding depression across a wide range of conditions are needed to enhance our ability to refine current guidelines (Hedrick and Fredrickson 2010; Frankham et al. 2011).

The crocodile lizard, *Shinisaurus crocodilurus*, offers a remarkable opportunity for experimental investigation into genetic rescue. This species is imperiled owing to small and fragmented populations resulting from anthropogenic activity, with only 950 individuals recorded in China in 2004 and fewer than 100 in Vietnam in 2014 (Huang et al. 2008; van Schingen et al. 2014). Despite its past abundance in the mountain ranges of Guangxi and Guangdong provinces in China (Zhang 1991), the population size has drastically declined from 6,000 individuals in 1978 to 950 in 2004 (Huang et al. 2008). Alarmingly, at least 7 small fragmented populations have become extinct. Subsequent studies revealed population declines of up to 70–80% in some of the main range populations by 2014 (Huang et al. 2014). Moreover, habitat fragmentation and disrupted gene flow have directly contributed to low genetic variability and increased inbreeding in this species (Ziegler et al. 2008; Huang et al. 2014; Xie et al. 2022). Consequently, as an integral aspect of an all-encompassing conservation management strategy, genetic rescue through the augmentation of genetic diversity holds the potential to salvage the remaining population fragments.

Captive breeding for the conservation of the crocodile lizard has been underway in China since the 1980s (Zhang 1991; Huang et al. 2008). In 2010, to address the issues of inbreeding and low genetic diversity in native captive breeding populations, an outbreeding program was launched in the Daguishan National Nature Reserve for Crocodile Lizards in Guangxi, China. Due to restricted access to Chinese populations other than the Daguishan population at that time, crossbreeding between Chinese and Vietnamese populations was established. Despite their geographical separation, Vietnamese populations exhibited some level of natural genetic exchange with Chinese populations, as documented in previous studies (Ziegler et al. 2008; Huang et al. 2014; Xie et al. 2022). Although this existing genetic exchange might suggest a lower risk of outbreeding depression, caution is warranted due to the potential challenges arising from their geographic isolation. Therefore, to assess and mitigate any risks associated with outbreeding depression, we deemed it advisable to conduct experimental crosses between Chinese and Vietnamese populations in captivity. This precautionary step preceded any consideration of translocating Vietnamese lizards into the wild population in China. By the end of 2017, the program had successfully produced 3 generations of outbred offspring along with a clear and accurate studbook. In this study, we aimed to evaluate the effect of augmenting gene flow between populations on the genetic diversity and fitness-related traits of crocodile lizards. Furthermore, we sought evidence of outbreeding depression in crocodile lizards by comparing the outbreeding population with the native breeding population reared under the same conditions. Outbreeding depression is anticipated to result in reduced fitness. Genetic incompatibilities, such as those arising from underdominance or epistatic interactions, can contribute to outbreeding depression (Ritland and Ganders, 1987; Lynch, 1991). Therefore, we examined critical fitness traits related to survival and reproductive output, and we also investigated the pattern of genetic introgression. Our goal was to investigate whether outcrossing led to a reduction in fitness and whether the hybrids exhibited genetic evidence of underdominance. Our research enabled us to assess the costs and benefits of genetic rescue by appropriate augmentation of gene flow between *S. crocodilurus* populations, and the implications for conservation management decisions.

## Materials and Methods

### Study species

The crocodile lizard, *S. crocodilurus*, is a semiaquatic lizard found in cool forests in southeast China and northeast Vietnam and is the only living member of the genus *Shinisaurus*. Life history traits of this species have been acquired in the captive population under semi-natural conditions, but not in the wild population. The crocodile lizard reaches sexual maturity at the age of 2.5 years and engages in mating from April to July. This viviparous lizard has a long gestation period spanning 9–12 months and shows a biennial reproductive cycle (Luo et al. 2022). Females typically yield a litter of 1–10 hatchlings, with larger females having heavier clutches (Zhang 1991; Huang et al. 2019; Li et al. 2019). The lifespan of the lizards in captivity can reach more than 10 years, with the longest recorded lifespan being more than 20 years in the Daguishan population.

### Experimental design

The captive populations of *S. crocodilurus* utilized in this study were established between 2010 and 2012 as part of an ex-situ breeding program in the Crocodile Lizard National Nature Reserve, Guangxi, China. The outbreeding population (VNCN) was initially established with 13 individuals, including 3 confiscated Vietnamese individuals (1 female and 2 males) and 10 Chinese individuals (8 females and 2 males). In subsequent generations, an additional 5 Chinese individuals (3 females and 2 males) were successfully introduced. On the other hand, the native captive breeding population (CNCN) was initially established with a group of 13 adults (8 females and 5 males) sourced from the local wild population of Daguishan, Guangxi (Supplementary Figure S1 and S2, Supplementary Table S1). In the second generation, an additional 2 Chinese individuals (1 female and 1 male) were introduced. The management of *S. crocodilurus* in captivity has been consistently implemented under the following conditions. We implemented a standardized mating system for both the CNCN and VNCN populations to streamline parentage determination. This involved pairing 1 male with 2 females. This approach aligns with the primary mating pattern observed in crocodile lizards in their natural habitat, where males tend to be polygynous, and females polyandrous, leading to multiple paternity among offspring from the same mother (Huang et al. 2015). The selection of sexually mature pairs adhered to 2 primary principles. Firstly, we aimed to minimize genetic relatedness as much as possible. Secondly, we ensured that individuals were of similar sizes. Our breeding observations indicated that significant size disparities can substantially impact mating success and potentially harm the individuals involved. To maintain experimental integrity, the 2 populations were housed in separate enclosures, with rigorous isolation measures in place to prevent any interaction with the local wild population. This study included all founders and their generations kept in captivity from 2010 to 2017 (Supplementary Table S1, Supplementary Figure S2). In addition, wild individuals from Guangxi and Vietnam were used to compare genetic characteristics with the VNCN and CNCN populations.

### Neutral SNP panel development and genotyping

We developed the SNP panel by mining the published re-sequencing raw reads of crocodile lizards (Huang et al. 2014). Firstly, we assembled the contigs according to the methods described in the original article. In order to define neutral loci, we implemented a stepwise approach by using Plink (v1.90b6.10, Purcell et al. 2007) to exclude SNPs potentially affected by selection pressures or other non-neutral forces: (1) SNPs within 100 kb downstream or upstream of a protein-coding gene were excluded; (2) SNPs that failed the Hardy–Weinberg Equilibrium (HWE) test (*q* < 0.05) were excluded. After this, we applied the following filtering steps to reduce linkage and exclude low-quality SNPs: (1) if the distance between 2 SNPs was less than 10 kb, we removed 1 SNP from the pair; (2) if 2 SNPs were in linkage disequilibrium (LD; *r*^*2*^ > 0.01), we randomly removed 1 SNP from the pair; (3) SNPs with low minor allele frequency (MAF < 0.3) were removed; (4) SNPs with a missing rate > 5% were excluded. Finally, we blasted all filtered SNPs to the reference genome by using Blast v2.2.28 (Johnson et al. 2008). SNPs surrounded by homozygous regions within 200 bp were excluded. Additionally, only SNPs with coverage > 0.75 and identity > 0.85 were retained. After filtering, a total of 463 putatively neutral SNPs (see featured summary of those SNPs in Supplementary Table S2) were obtained from 4,568,681 variant sites.

We randomly genotyped 100 neutral SNPs from the retained sequences to examine the consistency and effectiveness of the SNP panel. The primers were designed with Primer 3 online (v. 0.4.0, Untergasser et al. 2012). The SNPs were genotyped by multiplex PCR according to the methods of Chen et al. (2016) and sequenced on an Illumina X-10 platform (BioWing Applied Biotechnology Company, Shanghai). The FASTX Toolkit (v. 0.0.13) was used to de-multiplex and clean the raw reads. Subsequently, clean reads were mapped to target sequences by using the Burrows–Wheeler Alignment tool (BWA v.0.7.17, Li and Durbin 2009), and SNP calling was performed using SAMtools (v.1.19, Li et al. 2009). Of the 100 SNPs we genotyped, 98 SNPs were viable. The primer sequences of these 98 SNP markers are provided in Supplementary Table S3. We then genotyped the 98 SNPs using genetic data obtained from the saliva of 51 captive individuals collected in 2020 (Supplementary Table S1), along with the 33 local wild individuals from Guangxi, China. In addition, genotypes of 10 wild Vietnamese individuals were obtained from the published data of Xie et al. (2022) (Supplementary Figure S1). Individuals with a missing genotype rate greater than 20% were removed.

### Genetic variation analysis

We utilized the neutral SNP panel to assess genetic variation among individuals from the CNCN, VNCN, as well as wild Chinese and wild Vietnamese populations. We estimated the observed heterozygosity (*Ho*) and expected heterozygosity (*He*) of each site by Plink (--hardy) to compare genetic variation between different populations. The inbreeding coefficient (*F*_*IS*_ = 1 – *Ho*/*He*) was calculated for each site at HWE (*q* > 0.05). Pairwise Weir & Cockerham *F*_ST_ was calculated per site with VCFtools (v.0.1.13, Danecek et al. 2011). We then calculated one-way ANOVA with post hoc Tukey’s honestly significant difference (HSD) to examine the statistical significance of differences in average *Ho*, *He*, and *F*_*IS*_ between groups.

### Introgression analysis

We used the neutral SNP panel to examine how individual alleles across the genome were inherited after outcrossing in the VNCN family. We anticipated that if outcrossing results in substantial adaptation or depression, it may lead to non-neutral introgression between the 2 parental populations, either occurring rapidly or being impeded. Although the SNP panel used in this study is putatively neutral, these neutral loci may not be targets of selection themselves. Instead, these alleles can be carried along if they are linked to another region under selection, a phenomenon known as the selective sweep effect (Nielsen et al. 2005; Miller et al. 2012). We utilized BGC v1.03 (Gompert and Buerkle 2012) for the introgression analysis, and a detailed description of the methods can be found in the Supplementary material.

### Pedigree analyses

Pedigree analyses were based on the studbook for *S. crocodilurus* covering the period from 1 May 2010 to 31 December 2017 (Supplementary Table S1). We annually counted the population size and performed a linear model to estimate the population growth rate in Excel (2019). Survival analysis was implemented for the VNCN and CNCN populations. We first plotted the survival curve for the CNCN and VNCN populations by generation. We then fitted Cox regression models (Cox 1972) to evaluate the impact of different breeding management strategies on lizard survival. The estimation, statistical assessment, and plotting of survival curves were conducted using the R packages “survival” (Therneau 2022) and “survminer” (Kassambara et al. 2021) in the R software (R core Team 2019). Finally, we defined 4 organismal traits to describe the reproductive output of the VNCN and CNCN populations: (1) total number of offspring (clutch size, including stillbirth), (2) the rate at which live offspring were produced (alive rate), (3) the total mass of live offspring (total mass), and (4) the average mass of live offspring (average mass).

## Results

### Genetic variation

**Figure 1.**
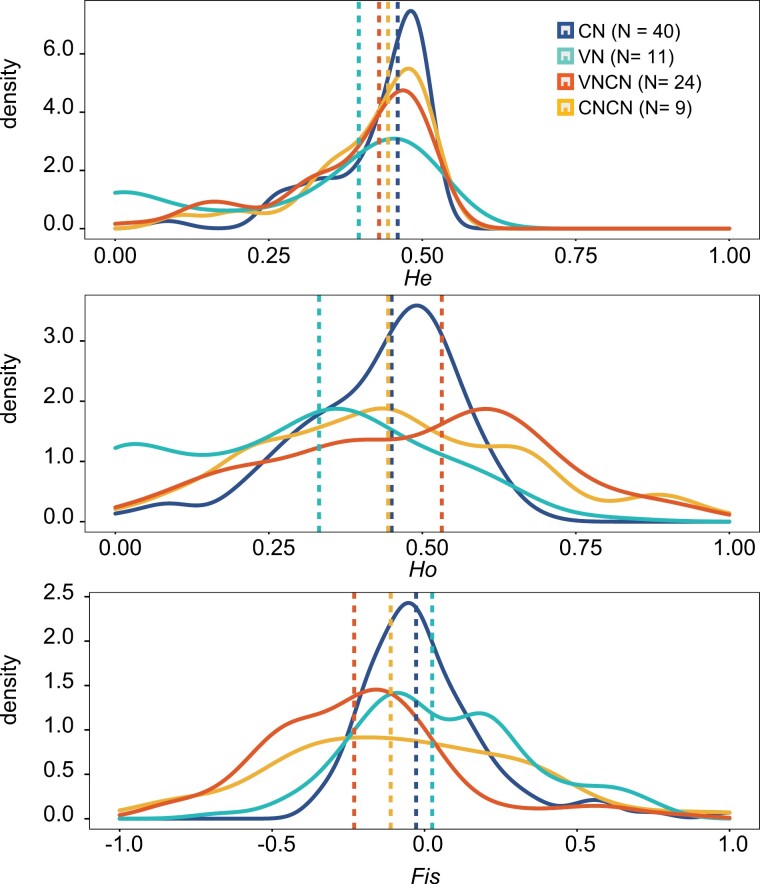
Genetic diversity statistics: expected heterozygosity (*He*), observed heterozygosity (*Ho*), and fixation index (*F*_*IS*_) for each population. The number of individuals from each population is indicated in brackets. The dashed lines indicate the median value. CN: All the wild-born individuals from China; VN: All the wild-born individuals from Vietnam; CNCN: The native breeding population; VNCN: The outbreeding population.

During our genetic variation analyses, 1 captive individual was removed due to an excessive missing rate of SNPs. Therefore, the final dataset consisted of 84 individuals (24 from the VNCN population, 9 from the CNCN population, 40 from the wild Chinese population, and 11 from the wild Vietnamese population), involving 98 neutral SNPs. The purebred CNCN population exhibited almost the same level of observed heterozygosity as the wild Chinese population (Figure 1, *Ho,* ANOVA*: P* > 0.05), with limited differentiation between them (Weir & Cockerham’s *F*_ST_, using 95% CI > 0 as significant: –0.014 [–0.048, 0.164]; Table 1). This suggests that the CNCN population captured the genetic diversity of the wild population quite well. We also observed a decrease in the degree of inbreeding (Figure 1, *F*_*IS*_). Compared with wild Chinese residents, wild Vietnamese residents had 18 private alleles and showed a median *F*_ST_ of 0.173 (Table 1). As expected, the outbred VNCN population maintained all of the wild Vietnamese’s private alleles and showed a much higher level of genetic diversity than both wild Chinese and Vietnamese populations (*Ho* in VNCN: 0.53 [0.06, 0.90], *Ho* in Chinese population: 0.45 [0.09, 0.6], *Ho* in Vietnamese population: 0.33 [0, 0.64], Figure 1), with moderate differentiation between outbred and purebred populations (median *F*_ST_: 0.106–0.126; Table 1).

**Table 1.** Pairwise *F*_ST_ (Weir & Cockerham’s) between 4 different populations of *Shinisaurus crocodilurus* based on 98 SNPs. The number of individuals from each population is indicated in brackets

	CN (40)	VN (11)	CNCN (9)
VN (11)	0.173 [–0.030, 0.729]		
CNCN (9)	–0.014 [–0.048, 0.164]	0.196 [–0.054, 0.811]	
VNCN (24)	0.106 [–0.016, 0.557]	0.126 [–0.032, 0.618]	0.119 [–0.036, 0.611]

CN: All the wild-born individuals from China; VN: All the wild-born individuals from Vietnam; CNCN: The native breeding population; VNCN: The outbreeding population. The median *F*_ST_ is displayed, and the number in square brackets are 95% confidence intervals for it.

### Genetic introgression of outbred population

The introgression analysis for the VNCN family found that almost all loci we examined showed no sign of being selected or coupled with selection. Only 1 locus significantly deviated from neutral introgression, providing evidence of a directional transition from Chinese to Vietnamese founders (*α* < 0; Supplementary Figure S3).

### Pedigree statistics

**Figure 2.**
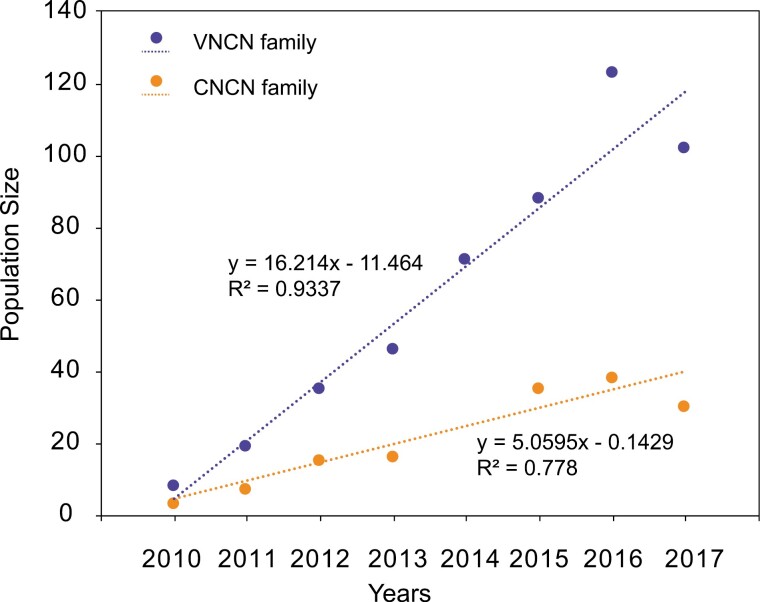
Variation in population size for the VNCN and CNCN population. The points represent the total number of all living individuals for the year and the dotted lines depict the linear fitting for each population. The *R*-squared represents the coefficient of determination.

The outbred VNCN family displayed a 3× higher growth rate (*r* = 16.21, *P* < 0.005, df = 14; Figure 2) than the purebred CNCN family (*r* = 5.06, *P* < 0.005, df = 14; Figure 2). The VNCN population reached a maximum population size (128 individuals) in 2016, while the CNCN population size plateaued as early as 2014 (37 individuals). By the end of 2017, the VNCN family had 3 generations with 107 surviving lizards, while the CNCN family had 2 generations with a much smaller population size of 30 lizards (Supplementary Figure S2, Figure 2).

**Figure 3.**
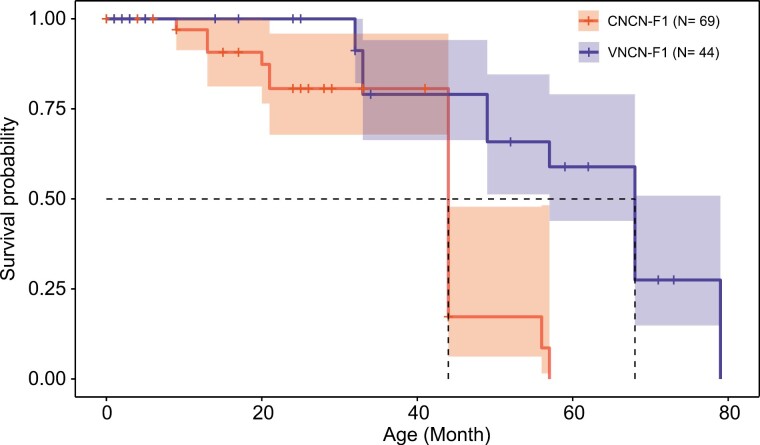
Survival curves with 95% confidence intervals for F1 generations of the CNCN population (CNCN-F1) and VNCN population (VNCN-F1). The dashed lines indicate the median survival time.

A Cox regression model (model goodness of fit: *z* = –4.108, *P* < 0.001) indicated that the F1 generation of the outbred population had a 5× higher survival rate than that of the native population (the hazard ratio of the VNCN-F1 relative to CNCN-F1 along with confidence intervals at 95%: 0.19 [0.09–0.42]; Figure 3). Meanwhile, both populations exhibited a decrease in survival rate with each successive generation. As the number of generations increased, our results revealed significant decreases in survival rates, ranging from 10 to 100s of folds (the hazard ratios for VNCN-F2 and VNCN-F3 relative to VNCN-F1 are 14.13 [6.15, 32.45] and 248.97 [47.19, 1313.72], respectively; the hazard ratio of CNCN-F2 relative to CNCN-F1 is 17.4 [3.321, 91.13]; Supplementary Figure S4). Nevertheless, reproductive output did not differ significantly between CNCN and VNCN families (*t*-test: *P* > 0.05; Supplementary Table S4).

## Discussion

Our study revealed that the captive populations of crocodile lizards show well-captured genetic diversity from the wild parental populations. Compared with the purebred CNCN population, the outbred pedigrees of the VNCN population exhibited a larger population size and a higher survival rate in the F1 generation. Although we did not find compelling evidence of outbreeding depression, we do not suggest that long-term monitoring is unnecessary. In general, our findings suggest that outbreeding management is a promising way to rescue the genetic diversity of this endangered lizard.

Outcrossing has resulted in 2 favorable outcomes for *S. crocodilurus.* The first is the increased population growth (Figure 2), considered an intuitive indicator of successful genetic rescue (Bell et al. 2019). Despite starting with the same number of initial founders, the outbred population was 3× larger and had one more generation by 2017 compared to the purebred population (Figure 2, Supplementary Figure S2). The immigration of Vietnamese individuals and the subsequent increase in population size directly contribute to the improvement of genetic diversity (VNCN; Figure 1), thereby increasing the potential for the population to maintain that genetic diversity. The second benefit of outcrossing was the positive effect on adult survival. We observed a significantly higher survival rate for the first generation of hybrids (Figure 3), which tend to live longer than the purebreds (~4.2 years on average for hybrids, compared with ~1.5 years on average for purebreds). This difference is unlikely to be caused by captive conditions, as all individuals were reared in the same captive facility with sufficient food, and all sexually mature individuals had equal opportunity to mate. Given that we found no significant difference in offspring output (hatchling size, offspring survival rate, and mass) between purebred and outbred populations (Supplementary Table S4), it is very likely that the difference in population size is due to the reduced number of reproductive events in the purebred population due to its shorter lifespan.

A major concern when conducting genetic rescue is the possibility of outbreeding depression (Edmands 2007; Frankham et al. 2011), especially when genetic donor populations meeting the criteria in the current guidelines are unavailable (Hedrick and Fredrickson 2010; Ralls et al. 2018). The lack of significant evidence for outbreeding depression in *S. crocodilurus* may be attributed to the close genetic relationship between Chinese and Vietnamese populations, as indicated by previous studies demonstrating considerable gene flow (Ziegler et al. 2008; Huang et al. 2014; Xie et al. 2022). The close genetic distance is associated with a low risk of outbreeding deficiencies (Edmands 1999) because severe genetic incompatibilities are unlikely to occur in closely related populations (Frankham et al. 2011). In addition, the introgression analysis did not detect any loci that showed evidence of underdominance (Supplementary Figure S3). Nevertheless, it’s worth noting that the results of this analysis may not be as conclusive as desired, primarily due to the restricted number of neutral loci used. Future research employing genome-wide analyses will offer enhanced insights into the effects of genetic rescue.

Therefore, we cannot exclude the possibility of delayed onset of outbreeding depression as the number of generations increases because heterosis is temporary and recombination can expose additional genetic incompatibilities over time (Tallmon et al. 2004). We detected a decrease in survival rate after the F1 generation in the VNCN population (Supplementary Figure S4). The crocodile lizard is a species characterized by a high level of inbreeding. During captive breeding, we have observed that females give birth to a substantial percentage of stillbirths (16.3%, Qiu et al. 2022). Given the inherent risk of inbreeding in crocodile lizards and the limited number of founders (only 13 for each population), the cumulative effects of inbreeding can be severe. However, it is difficult to determine whether the decrease in survival rate is due to subsequent inbreeding or the risk of outbreeding depression. Current guidelines are primarily derived from studies limited to the F1 and F2 generations (Tallmon et al. 2004; Whiteley et al. 2015). A long-term evaluation of the impact of genetic rescue is necessary to determine the duration of the initial positive effect of genetic rescue and any possible negative effects arising over time (Bell et al. 2019).

Although the potential for outbreeding depression cannot be entirely ruled out at this stage, our results strongly suggest genetic rescue. The outcomes of our study provide a compelling demonstration of genetic rescue in the endangered crocodile lizard, underscoring the constrained but valuable application of genetic rescue strategies in modern conservation practices (Ralls et al. 2018; Fitzpatrick et al. 2023). We monitored the population-level fitness and genetic outcomes of outbreeding across multiple generations, contrasting these results with those of the purebred population in captive conditions. This approach allowed us to establish a clear causal relationship between heterosis (elevated fitness of F1 hybrids compared to their parents) and its impact on overall fitness. However, addressing these crucial questions, such as the potential adverse effects of introduced gene flow with increasing generations of crossbreeding, the maintenance of crossbreeding benefits in the natural environment, and the precise control of gene flow amounts, requires ongoing monitoring efforts. In general, striking a balance between the potential benefits and risks of genetic rescue is a complex issue, and an increasing number of decision-support tools serve as guidance (Frankham et al. 2017; Ralls et al. 2018; Liddell et al. 2021). We advocate for enhanced pre/post outbreeding monitoring to address this issue more comprehensively. The insights gained from such studies make a significant contribution to evidence-based conservation decision-making.

## Supplementary Material

Supplementary material can be found at https://academic.oup.com/cz.

zoae003_suppl_Supplementary_Figures_S1-S4

zoae003_suppl_Supplementary_Table_S1

zoae003_suppl_Supplementary_Table_S2

zoae003_suppl_Supplementary_Table_S3

zoae003_suppl_Supplementary_Table_S4

## Data Availability

The SNP dataset analyzed in this study is available from Figshare (DOI: 10.6084/m9.figshare.21646889).
