# Supplementary material for: Outbreeding management offers the promise of genetic rescue for an endangered lizard
Source: Curr Zool. 2024 Feb 27;70(6):721–7. doi: 10.1093/cz/zoae003 (PMC11634679; doi:10.1093/cz/zoae003)

**Supplementary information for:**

Outbreeding management offers the promise of genetic rescue for an endangered lizard

**Supplementary methods**

***Introgression analysis***

We examined the patterns of inheritance at individual loci using BGC v1.03. This Bayesian genomic cline model detects movement of genetic materials from one genomic background to another between two parental populations. A null model of neutral introgression was then generated via parametric procedure (outlined in Gompert & Buerkle 2009), which uses allele frequencies directly from the parental populations to calculate expected probabilities of locus-specific genotypes. The parametric procedure is recommended for multiallelic loci that do not exhibit fixed differences between the parental populations. This null model of neutral introgression was then compared with the empirical regression using log-likelihood ratios. Deviations between the empirical and null models are indicative of non-neutral introgression. Two key parameters were used to describe the result: cline parameter α designates an increase (positive value) or decrease (negative value) in the probability of ancestry from one parent to the other for a locus, whereas cline parameter β specifies an increase (negative value) or decrease (positive value) in the strength of the barrier effect to gene flow from one parent to the other (Gompert & Buerkle 2012).

For this study, parental populations were defined as domestic founders (all Chinese founders for VNCN and their pure progeny, N = 9, Table S1) and migrants (all Vietnamese founders for VNCN and their pure progeny, N = 10, Table S1). And the admixed population was characterized with some VNCN progeny (N = 24, Table S1). We ran three independent MCMC chains with 100,000 steps and discarded the first 75,000 steps as burn-in. Samples were recorded from the posterior distribution every 25 steps. We then combined three chains after inspecting the convergence of MCMC outputs. Loci with α or β values where 95% CIs significantly deviated from zero were designated as gene flow outlier loci with exceptional introgression. The results were plotted using the R package ‘ClineHelpR’ (available at https://github.com/btmartin721/ClineHelpR) in the R software (R core Team, 2019).

**Figure S1. Population distribution of S. *crocodilurus* in this study.** Two remnant wild populations, Guangxi (GX) and Vietnam (VN), are represented by black points. The asterisk depicts the location of the nature reserve where the breeding program is carried out.

**Figure S2.** **Pedigree tree depicting the 297 captive individuals:** (a) 184 outbred individuals (depicted with black lines, VNCN) and 13 purebred Vietnamese pedigrees from one Vietnamese couple (depicted with gray lines, VNVN). All individuals descended from 13 wild lizards, of which 3 Vietnamese individuals are illustrated with red text; (b) 78 purebred Chinese individuals (depicted with black lines, CNCN) with 13 founders. The individuals are represented as dots and the individuals with their studbook identification number are founders for this population. The lines connecting dots indicate reproductions.

**Figure S3. Scatterplots show values of the genomic cline center parameter (α) and the genomic cline rate parameter (β) for VNCN family.** Each point represents a diagnostic locus. Gray dots are neutral loci (non-outliers), and blue dot represent locus with parameter α significantly deviated from zero.

**Figure S4. Survival curves with 95% confidence intervals for each generation of CNCN and VNCN families.** The number of individuals for each group are indicted in brackets.


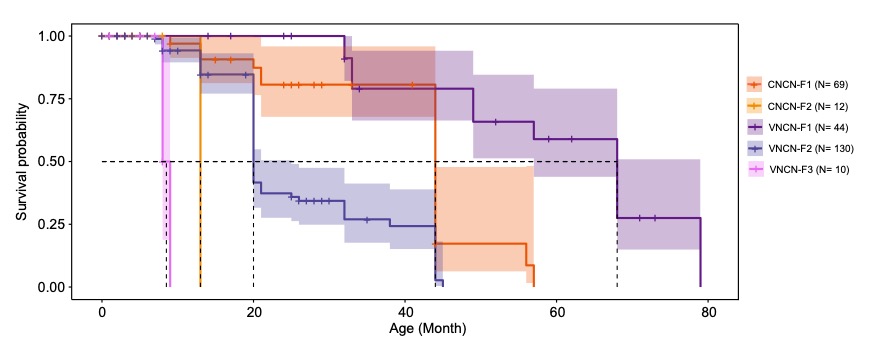

Supplement: zoae003_suppl_Supplementary_Figures_S1-S4 [file zoae003_suppl_supplementary_figures_s1-s4.docx]
